# Lyophilized Maqui (*Aristotelia chilensis*) Berry Induces Browning in the Subcutaneous White Adipose Tissue and Ameliorates the Insulin Resistance in High Fat Diet-Induced Obese Mice

**DOI:** 10.3390/antiox8090360

**Published:** 2019-09-01

**Authors:** Viviana Sandoval, Antoni Femenias, Úrsula Martínez-Garza, Hèctor Sanz-Lamora, Juan Manuel Castagnini, Paola Quifer-Rada, Rosa Maria Lamuela-Raventós, Pedro F. Marrero, Diego Haro, Joana Relat

**Affiliations:** 1 Department of Nutrition, Food Sciences and Gastronomy, School of Pharmacy and Food Sciences, Food Torribera Campus, University of Barcelona, E-08921 Santa Coloma de Gramenet, Spain; 2 Institute of Nutrition and Food Safety of the University of Barcelona (INSA-UB), E-08921 Santa Coloma de Gramenet, Spain; 3 Applied Mycology Unit, Food Technology Department, University of Lleida, UTPV-XaRTA, Agrotecnio, E-25198 Lleida, Spain; 4 Facultad de Ciencias de la Alimentación, Universidad Nacional de Entre Ríos, Tavella 1450, 3200 Concordia, Argentina; 5 Department of Endocrinology & Nutrition, CIBER of Diabetes and Associated Metabolic Diseases, Biomedical Research Institute Sant Pau, Hospital de la Santa Creu i Sant Pau E-08041 Barcelona, Spain; 6 CIBER Physiopathology of Obesity and Nutrition (CIBER-OBN), Instituto de Salud Carlos III, E-28029 Madrid, Spain; 7 Institute of Biomedicine of the University of Barcelona (IBUB), E-08028 Barcelona, Spain

**Keywords:** anthocyanins, browning, carbohydrate-responsive element binding protein b, delphinidin, fibroblast growth factor 21, high-fat diet, maqui berry, white adipose tissue

## Abstract

Maqui (*Aristotelia Chilensis*) berry features a unique profile of anthocyanidins that includes high amounts of delphinidin-3-O-sambubioside-5-O-glucoside and delphinidin-3-O-sambubioside and has shown positive effects on fasting glucose and insulin levels in humans and murine models of type 2 diabetes and obesity. The molecular mechanisms underlying the impact of maqui on the onset and development of the obese phenotype and insulin resistance was investigated in high fat diet-induced obese mice supplemented with a lyophilized maqui berry. Maqui-dietary supplemented animals showed better insulin response and decreased weight gain but also a differential expression of genes involved in de novo lipogenesis, fatty acid oxidation, multilocular lipid droplet formation and thermogenesis in subcutaneous white adipose tissue (scWAT). These changes correlated with an increased expression of the carbohydrate response element binding protein b (*Chrebpb*), the sterol regulatory binding protein 1c (*Srebp1c*) and Cellular repressor of adenovirus early region 1A–stimulated genes 1 (*Creg1*) and an improvement in the fibroblast growth factor 21 (FGF21) signaling. Our evidence suggests that maqui dietary supplementation activates the induction of fuel storage and thermogenesis characteristic of a brown-like phenotype in scWAT and counteracts the unhealthy metabolic impact of an HFD. This induction constitutes a putative strategy to prevent/treat diet-induced obesity and its associated comorbidities.

## 1. Introduction

Stimulation of the brown adipose tissue (BAT) and the induction of browning in white adipose tissue (WAT) as a strategy against obesity and its associated metabolic complications has generated growing interest in recent years. This interest is based on the ability of both BAT and browned-WAT to increase energy expenditure (EE) mainly through fatty acid consumption [1,2], and their pivotal role in the control of energy homeostasis in mammals [3,4]. In addition to classical brown adipocytes located in BAT, thermogenic adipocytes with similar characteristics can be found within white adipose tissue (WAT) [3]. These brite/beige adipocytes are metabolically and phenotypically similar to brown adipocytes and can actively contribute to increasing whole-body EE. Specifically, brite/beige adipocytes show a multilocular phenotype and express genes closely related to BAT metabolism (Ucp1 as a marker of its thermogenic capacity in addition to genes implied in de novo lipogenesis (DNL), fatty acid oxidation (FAO), lipolysis, etc.).

Recent evidence shows that the activation of BAT and the induction of browning in WAT can be induced by cold acclimation but also by nutritional inputs under different signaling cascades [2,5,6,7]. Cold is a classic activator of BAT and of beige adipocyte development and function [5,8]. Regarding nutritional inputs, we recently demonstrated that low-protein diets and the cooked-tomato sauce called “sofrito” are able to induce Ucp1 expression in WAT, thus indicating a browning phenotype [6,7]. In the same way, other authors published that high-fat diets, bioactive compounds and prebiotics can also induce browning in WAT [9,10,11,12,13,14]. 

Part of the cold-induced metabolic profile in BAT is regulated by the stimulation of carbohydrate-responsive element binding protein b (ChREBPb) through the Ak strain transforming/protein kinase 2 (AKT2) activity [15]. Besides ChREBP, fibroblast growth factor 21 (FGF21) has shown beneficial effects on glucose/lipid homeostasis and body weight control among other mechanisms by increasing energy expenditure (EE) and inducing browning and UCP1 overexpression in adipose tissues [6,16,17,18,19], as well as by promoting the insulin-dependent glucose uptake, mitochondrial biogenesis, and adiponectin secretion in adipocytes [20,21]. In this case, it has been widely demonstrated that FGF21 activity and/or signaling respond to nutritional challenges [22].

Anthocyanidin-rich berries have been proposed for the treatment and prevention of several disorders, including obesity-related metabolic disorders [23,24,25,26,27,28,29,30,31,32] but little is known about the molecular mechanisms underlying their beneficial effects. Maqui (*Aristotelia chilensis*) is a native Chilean berry with a unique anthocyanins profile that includes delphinidin-3-O-sambubioside-5-O-glucoside and delphinidin-3-O-sambubioside as the main phenolic compounds [33]. Besides its antioxidant activity, different preparations of maqui have shown positive effects on fasting glucose and insulin levels in humans and murine model of type 2 diabetes and obesity [34,35,36,37] and delphinidin-3-sambubioside-5-glucoside has been described as the responsible for hypoglycemic activity in in vivo models [36].

With the global aim of deepening the knowledge of the molecular mechanisms responsible for the metabolic effects of maqui and in some way of the anthocyanidin-rich foods, we investigated the effect of a lyophilized maqui berry preparation on the onset and progression of the diet-induced obesity (DIO) in mice subjected to high-fat diet (HFD) for 16 weeks. We studied the impact of maqui in the metabolic profile of the obese phenotype. We focused on the adipose tissue metabolic phenotype because of its role on the progression of obesity and as a major target to counteract the onset and development of this pathology and its metabolic-associated diseases such as insulin resistance. We analyzed specifically the subcutaneous WAT (scWAT) because growing evidence suggests that this depot is protective to metabolic health while visceral is detrimental [38,39,40,41,42,43]. 

Globally, our results highlight the potential role of maqui in the treatment of diet-induced obesity and insulin resistance. Further studies will be needed to identify the effects of maqui on healthy population and the impact of its regular consumption as part of a healthy dietary pattern such as the Mediterranean diet. 

## 2. Materials and Methods 

### 2.1. Anthocyanins Determinations by UPLC-DAD

For sample preparation, 0.1 g of maqui was extracted with 5 mL of a mixture of water and ethanol 80:20 (*v/v*). The extraction was repeated 3 times to increase extractability of the anthocyanidins. The extract was evaporated under vacuum to remove the ethanol and reconstituted to a final volume of 10 mL with MilliQ water. This procedure was done in triplicate. Finally, the sample was filtered through a 0.22 µm PTFE membrane filter into an amber vial for UPLC analysis.

The quantification and identification of anthocyanins was done using a Waters Acquity Ultra Performance Liquid Chromatography H class (Waters Corp, Milford MA, USA) coupled to Photodiode Array (PDA) detector (Waters Corp, Milford MA, USA). The chromatographic separation was performed by an Aquity BEH C18 column 2.1 mm × 100 mm, 1.7 µm. The chromatographic method proposed by Andrés-Lacueva et al. (2005) adapted to the UPLC system was used [44]. Briefly, the injection volume was 10 µL and the gradient elution was performed with water/5% formic acid (*v/v*) (A) and 100% acetonitrile (B) at a constant flow rate of 0.75 mL/min. A decreasing linear gradient of solvent A was used. Separation was carried out in 11 min under the following conditions: 0 min, 98% A; 6 min, 95% A; 9 min, 90% A; 9.1 min, 20% A; 9.3min, 20% A; 9.4 min, 92% A, 11 min, 92% A. 

The column was equilibrated for 6 min prior to each analysis. Each maqui anthocyanin was quantified at *λ* = 520 nm using a calibration curves with pure standard purchased in Extrasynthese S. A. (Delphinidin-3-O-sambubioside-5-O-glucoside, delphinidin-3-O-sambubioside, cyanidin-3-O-sambubioside-5-O-glucoside, cyanidin-3-O-glucoside and cyanidin-3-O-sambubioside), and the identification was made by comparing the retention times of the chromatographic peaks with the retention time of the pure phenolic standards. Results were expressed as millimoles of anthocyanins per kilogram of maqui (mmol/kg).

### 2.2. Animal Procedures—Dosage Regimen

Animal procedures were approved by the Animal Ethics Committee of the University of Barcelona (CEEA-173/18). C57BL/6J littermates’ male mice (*n* = 23) were housed in a temperature-controlled room (22 ± 1 °C) on a 12-h/12-h light/dark cycle and were provided free access to commercial rodent chow and tap water prior to the experiments. When animals were four-week-old it was confirmed that all animals were normoglycemic before being randomly assigned into two experimental groups (HFD and HFD supplemented with maqui (HFDM)). Both groups were fed a diet of 45% fat-derived calories (HFD) (D12451, Research Diets) for 16 weeks supplemented or not with lyophilized maqui. HFD group (*n* = 9) had free access to HFD diet and filtered-tap water and HFDM (*n* = 14) group had free access to HFD diet and filtered-tap water supplemented with maqui (20 mg of lyophilized maqui/mL of filtered-tap water). To prepare the supplemented water, 1 g of the lyophilized maqui was added to 50 mL of tap-filtered water. This mixture was prepared extemporaneously every two days to prevent the oxidation of maqui bioactive compounds.

The dosage regimen of maqui was calculated according to the polyphenol intake recommended as beneficial by the Predimed Study (820 mg in a human diet of 2300 kcal) [45,46]. Mice intake is around 10–15 kcal per day, which means 4–5 mg of polyphenols per day scaling-down the recommended beneficial quantity in humans. Table 1 shown that 1g of lyophilized maqui provides 45 mg of anthocyanins. The dose of maqui was adjusted to achieve 4 mg of polyphenols per day, considering that mice take 2–3 mL of water/day. The nutritional composition of the lyophilized maqui used (Maquiberry, Native for Life, Chile) is indicated in the supplemental information (Table S1). 

During the 16-week nutritional intervention, food and beverage intake were recorded every two days and body weight twice a week. At the end of the nutritional intervention, the animals were euthanized. Blood was extracted by intracardiac puncture, and serum was obtained by centrifugation (1500 rpm, 20 min). Epididymal WAT (eWAT), scWAT and BAT were isolated, immediately snap-frozen and stored at −80 °C for future analysis.

### 2.3. RNA Isolation and Quantitative RT-PCR

Total RNA was isolated from frozen tissues using TRI Reagent™ Solution (AM9738, Thermo Fisher Scientific, Waltham, USA), followed by DNaseI treatment (K2981, Thermo Fisher Scientific, Waltham, USA). cDNA was synthesized from 1 μg of total RNA using the High-Capacity cDNA Reverse Transcription Kit (4368814, Thermo Fisher Scientific, Waltham, USA). Relative mRNA levels were measured by quantitative PCR (qPCR) using SYBR™ Select Master Mix for CFX (4472942, Thermo Fisher Scientific, Waltham, USA). 18S and B2M were used as housekeeping genes. The sequences of the primers used in qPCR are presented in Table S2. Results were obtained by the relative standard curve method, and values were referred to the HFD group.

### 2.4. Glucose Tolerance Test (GTT) and Insulin Tolerance Test (ITT)

Mice were fasted for 6 h in the morning, and then injected intraperitoneally (i.p.) with 1.5 g of glucose (Sigma)/kg mouse (GTT) or 0.5 UI of insulin solution (Sigma)/kg mouse (ITT). Blood samples were collected from the tail vein, and glucose levels were measured using a glucometer (Glucocard SM, Menarini, Florence, Italy) prior to the i.p. injection and at 30, 60 and 120 min postinjection. GTT was performed 14 weeks after the beginning of maqui supplementation and ITT on week 15th.

### 2.5. Histological Analysis

For the histological analysis, pieces of scWAT of each animal were fixed in 10% formalin (Sigma) and embedded in paraffin. Afterwards, 4 µm-thick sections were cut and stained with hematoxylin and eosin (H&E). Images were acquired using a Digital Upright Microscope BA310 Digital and a Moticam 2500 camera. The selection of the test objects was performed according to color and choosing the same limits for binarization for all images. At least three pictures from different regions of each cut were taken.

### 2.6. Data Analysis/Statistics

GraphPad Prism version 8.02 (GraphPad, San Diego, CA, USA) was used to perform the statistical analyses. Two tailed Student’s Test with Welch’s correction when not equal SDs can be assumed was used to determine significant differences among experimental groups. The statistical analysis of body weight progression, body weight/calorie intake and curves of GTT and ITT was performed by 2 factor ANOVA with pot-hoc test (Bonferroni’s). In all cases, *p*-value < 0.05 was considered statistically significant. All data are expressed as the mean ± SEM.

## 3. Results

### 3.1. Maqui Anthocyanin Content

The levels of anthocyanins determined in the lyophilized maqui used in our experimental approach (Maquiberry, Native for Life, Chile) (Table 1) were similar to those reported previously [47,48,49,50]. In terms of the total content of anthocyanins, the lyophilized maqui used in this work has an extremely high content (45,052 mg/kg = 4.5%) compared to other similar products like a Freeze-Dried Whole Blueberry (2432 mg/kg = 2.4%) [51] or a dried raspberry solids (750 mg/kg = 0.75%) [52]. The predominant (80%) anthocyanins were delphinidin-3-O-sambubioside-5-O-glucose and delphinidin-3-O-sambubioside, as shown in Table 1 and Figure S1 (Supplemental Materials).

### 3.2. Maqui Dietary Supplementation Reduces HFD-Induced Body Weight Gain and Improves Insulin Sensitivity in Mice

**Figure 1:**
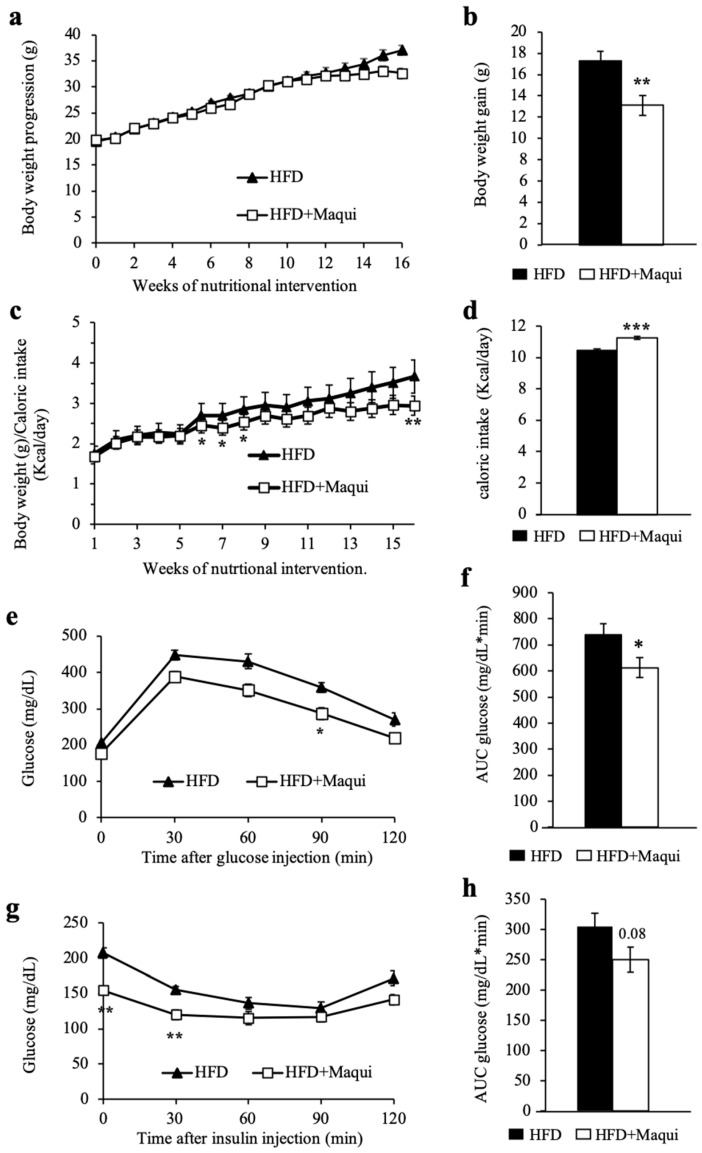
Maqui dietary supplementation reduces HFD-induced body weight gain and improves insulin sensitivity in mice. (**a**) Body weight progression (g) for the 16-week nutritional intervention with maqui. Body weight was recorded twice a week. The graph represents the mean ± SEM of weekly increments in both experimental groups. (**b**) Total body weight increment in grams after 16-week nutritional intervention with maqui. The graph represents the mean ± SEM of the total body weight increment in both experimental groups. (**c**) Body weight (g) related to calorie intake (kcal) per week for the 16-week nutritional intervention with maqui. Calorie intake was calculated based on the energy density of the HFD and the amount of food consumed daily. In HFDM mice, the kcal from maqui were also added. The graph represents the mean ± SEM of the weekly body weight increase related to calorie intake in both experimental groups. (**d**) Calorie intake for both experimental groups during the 16-week nutritional intervention. The graph represents the mean ± SEM. (**e**) GTT curve showing plasma glucose levels after i.p. administration of glucose (1.5 g/kg b.w.) in HFD and HFDM mice after 14 weeks of maqui supplementation. (**f**) AUC of glucose levels in GTT. (**g**) ITT curve showing plasma glucose levels after i.p. administration of insulin (0.5 UI/kg b.w.) in HFD and HFDM mice after 15 weeks of maqui administration. (**h**) AUC of glucose levels in ITT. Data from GTT and ITT are presented as the mean ± SEM. * *p* < 0.05, ** *p* < 0.01, *** *p* < 0.001 versus the HFD group.

C57BL6/J mice fed an HFD for 16 weeks put on weight (Figure 1a,b) and displayed glucose intolerance (Figure 1e,f) and insulin resistance (Figure 1g,h). Comparing both experimental groups revealed that mice fed HFD supplemented with maqui (HFDM) showed an attenuated progression in body weight (Figure 1a–c), even the animals were hyperphagic, and their daily caloric intake was higher (Figure 1d). Concretely, the HFDM animals put on less weight after the 16 weeks of nutritional intervention (Figure 1b). Even though no statistical differences were observed in the body weight progression (Figure 1a), there were when the ratio between body weight and caloric intake was calculated (Figure 1c). These data indicated that, in some way, there is an increased energy expenditure in these animals. 

Regarding the insulin/glucose responsiveness, HFDM mice shown a significant reduction in fasting glucose at Week 15 of the nutritional intervention (207.6 ± 5.5 vs. 166.5 ± 6.1, *p* = 2.7 × 10^−5^). It is worth highlighting that, after the glucose injection, the HFDM animals displayed a better glucose curve (Figure 1e) that corresponded to a significant reduction of the area under the curve (AUC) of the GTT (Figure 1f, *p* < 0.05). In the case of the ITT, the curve of glucose after the insulin injection showed significantly lower levels of glucose at the first time-points (Figure 1g). These differences were minimized but maintained over time, even though they did not reach statistical significance (Figure 1g). Finally, the AUC of the ITT showed a clear tendency to a reduction in HFDM (Figure 1h, *p* < 0.08). Although some of the data are not significant individually, altogether they indicate an improvement on glucose tolerance and insulin sensitivity after 16 weeks of maqui dietary supplementation. 

### 3.3. Maqui Dietary Supplementation Induces a Multilocular Phenotype in scWAT

**Figure 2:**
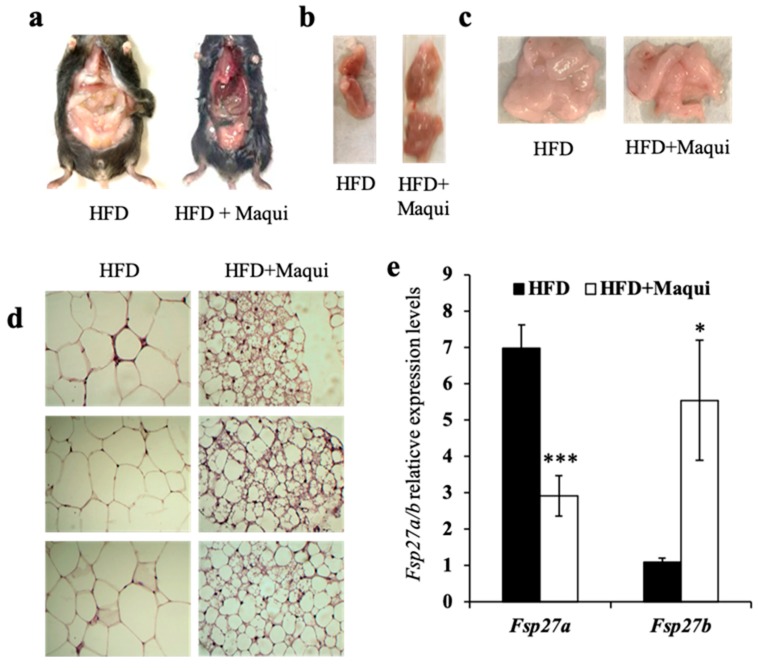
Maqui dietary supplementation induces a multilocular phenotype in scWAT. (**a**) Representative pictures of HFD (*n* = 9) and HFDM (*n* = 14) mice after 16 weeks of nutritional intervention. HFDM mice are leaner and show fewer white fat depots. (**b**) Representative pictures of interscapulum BAT of HFD and HFDM mice. For HFDM mice, the BAT depot is larger than the one from HFD mice. (**c**) Representative pictures showing scWAT depots of HFD and HFDM mice. (**d**) Representative hematoxylin and eosin (H&E)-stained scWAT sections from HFD and HFDM (40× magnification). Some multilocular adipocytes are revealed in scWAT of HFDM, but none were seen in the HFD animals. (**e**) *Fsp27a* and *Fsp27b* mRNA levels were measured by qRT-PCR in scWAT of HFD and HFDM mice. Bars represent the relative mRNA levels of both genes in the two experimental conditions in scWAT normalized by the *B2M* gene as housekeeping gene. Data are presented as the mean ± SEM. * *p* < 0.05, *** *p* < 0.001 versus the HFD group.

HFDM mice were leaner than their littermates (Figure 1a and Figure 2a) and had more BAT and less WAT (Figure 2b,c). Because of the healthier profile observed in HFDM mice regarding body weight and insulin resistance, we hypothesized that maqui could be exerting its effects through the induction of browning in scWAT. The H&E staining of scWAT revealed that maqui supplementation induced the transition of unilocular adipocytes to multilocular ones (Figure 2d). No differences in other WAT depots due to maqui supplementation were observed in any analysis performed.

In the context of browning, recent publications have shown the relevance of fat-specific protein 27 (FSP27) isoforms in the phenotype of unilocular or multilocular lipid droplets. FSP27 is considered to be, at least in part, the protein responsible for the formation and growth of lipid droplets (LDs). Two isoforms have been described with different expression patterns and functions. Fsp27a is expressed in WAT, where it promotes the formation of large LDs. By contrast, BAT expresses the Fsp27b isoform that contributes to the ensemble of smaller LDs [53,54]. To confirm the brown-like phenotype of scWAT in HFDM mice, the mRNA levels of both *Fsp27* isoforms were measured. The results indicated that the dietary maqui supplementation causes a shift in the expression pattern of Fsp27. The scWAT depot of HFDM mice showed a significant reduction in the levels of *Fsp27a* and a significant induction of *Fsp27b* expression (Figure 2e), thus reinforcing the idea that maqui causes a brown-like phenotype in the scWAT.

### 3.4. Maqui Induces the Expression of Genes from de Novo Lipogenesis, Fatty Acid Oxidation, Thermogenesis and Browning in scWAT

**Figure 3:**
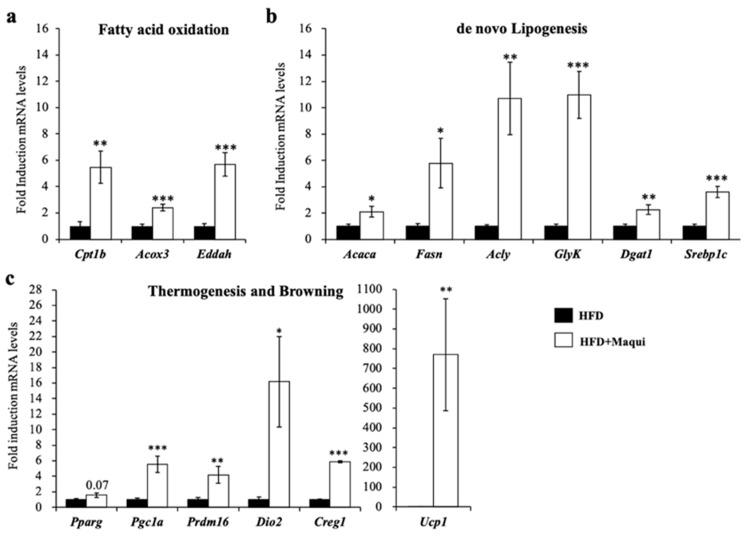
Maqui induces the expression of genes from de novo lipogenesis, fatty acid oxidation, thermogenesis/browning in scWAT. The relative mRNA levels of characteristic genes of (**a**) mitochondrial and peroxisomal FAO (*Cpt1b, Acox3, and Ehhadh*), (**b**) DNL (*Acaca, Acly, Fasn, GlyK, Dgat1 and Srebp1c*) and (**c**) thermogenesis and browning (*Ucp1, Type 2 Iodothyronine Deiodinase (Dio2), PR-Domain Zinc Finger Protein 16 (Prdm16), Peroxisome proliferator-activated receptor gamma (Pparg), Peroxisome proliferator-activated receptor gamma coactivator 1 alpha (Pgc1a) and Cellular repressor of adenovirus early region 1A–stimulated genes 1 (CREG1)*) were measured using qRT-PCR in scWAT of HFD and HFDM mice. Bars represent the fold induction in the mRNA levels versus the HFD animals that are considered the control group, which produces an arbitrary value of 1. Data are presented as the mean ± SEM. * *p* < 0.05, ** *p* < 0.01, *** *p* < 0.001 versus the HFD group.

As shown in Figure 3, in the scWAT of HFDM mice, the mRNA levels of genes related to mitochondrial (carnitine palmitoyl transferase 1b (Cpt1b)) and peroxisomal (Acyl-CoA oxidase 3 (Acox3), Enoyl-Coenzyme A, and Hydratase/3-Hydroxyacyl Coenzyme A Dehydrogenase (Ehhadh)) FAO (Figure 3a), DNL (Acetyl-CoA Carboxylase Alpha (Acaca), ATP Citrate Lyase (Acly), Fatty acid synthase (Fasn), Diacylglycerol Acyltransferase I (Dgat1), Sterol regulatory binding protein 1c (Srebp1c) and Glycerol Kinase (GlyK)) (Figure 3b) and thermogenesis/browning (Ucp1, Type 2 Iodothyronine Deiodinase (Dio2), PR-Domain Zinc Finger Protein 16 (Prdm16), Peroxisome proliferator-activated receptor gamma (Pparg), Peroxisome proliferator-activated receptor gamma coactivator 1 alpha (Pgc1a) and Cellular repressor of adenovirus early region 1A–stimulated genes 1 (Creg1)) (Figure 3c) increased, suggesting that the scWAT of the HFDM mice were metabolically closer to BAT than WAT. We also analyzed epididymal WAT (eWAT) and BAT. While eWAT did not show any feature of browning (data not shown), BAT showed an increment of size (Figure 2b) but no induction of Ucp1 was observed (data not shown).

### 3.5. Maqui Induces the Expression of Chrebpa, Chrebpb and Glut4 in scWAT

**Figure 4:**
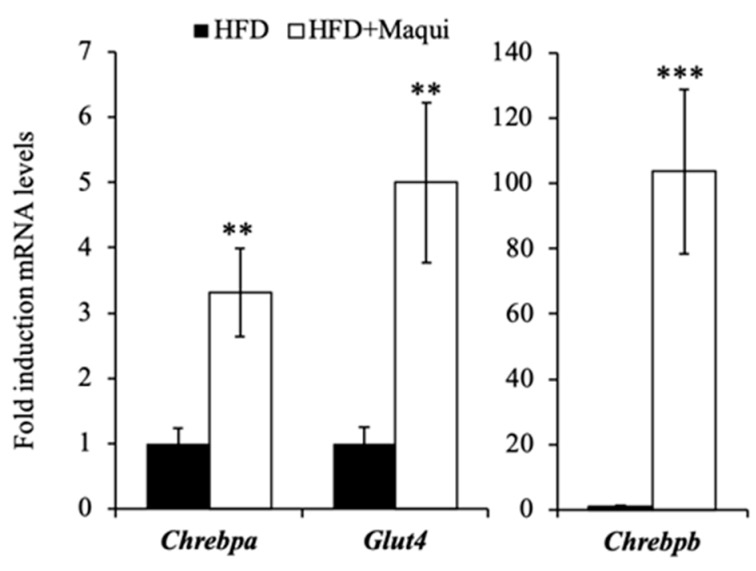
Maqui induces the expression of *Chrebpa, Chrebpb* and *Glut4* in scWAT. The relative mRNA levels of *Glut4, Chrebpa* and *Chrebpb* were measured by qRT-PCR in scWAT of HFD and HFDM mice. Bars represent the fold induction in the mRNA levels versus the HFD animals that are considered the control group and assigned an arbitrary value of 1. Data are presented as the mean ± SEM. ** *p* < 0.01, *** *p* < 0.001 versus the HFD group.

As mentioned above, part of the cold-induced metabolic profile in BAT is regulated by the stimulation of ChREBPb through the AKT2 activity [15]. In WAT, the expression of Chrebp in adipose tissue is regulated by GLUT4-mediated glucose uptake that activates the ChREBPa isoform, which in turn induces the expression of Chrebpb, an alternative-promoter transcribed isoform. Chrebpb expression in human adipose tissue predicts insulin sensitivity, and its induction has been highlighted as an effective strategy for preventing and treating obesity-related metabolic dysfunction and type 2 diabetes [55,56,57]. Globally, ChREBP is considered the major regulator of DNL in adipose tissue [58]. To analyze the putative role of ChREBPb and GLUT4 in the induction of the metabolic changes observed in scWAT due to maqui supplementation, the mRNA levels of Glut4, Chrebpa and Chrebpb were evaluated. The data show that maqui increases the expression of these three genes (Figure 4), thus providing insight into the molecular mechanism through which maqui induces a brown-like phenotype in scWAT.

### 3.6. Maqui Increases the Expression of Adiponectin and FGF21 and the FGF21 Signaling in the scWAT

**Figure 5:**
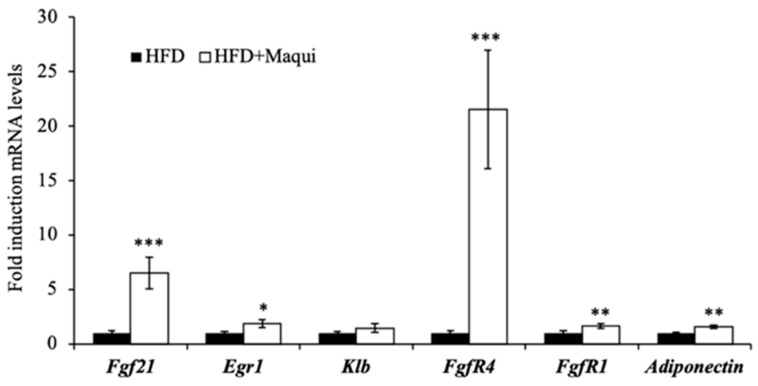
Maqui induces the expression of *Fgf21, Fgf21 receptors* and FGF21 signaling markers in scWAT. The relative mRNA levels of *Fgf21, Fgf21R1, FGFR4, KLB, Adiponectin* and *Egr1* were measured by qRT-PCR in scWAT of HFD and HFDM mice. Bars represent the fold induction in the mRNA levels versus the HFD animals that are considered the control group and assigned an arbitrary value of 1. Data are presented as the mean ± SEM. * *p* < 0.05, ** *p* < 0.01, *** *p* < 0.001 versus the HFD group.

Obesity is considered an FGF21-resistant state usually due to a downregulation of the fibroblast growth factor receptor (FGFR) and/or its co-receptor β-klotho (KLB) that impairs FGF21 signaling [59,60]. To analyze the effect of maqui berry supplementation on the FGF21 signaling, the mRNA levels of Fgf21 itself, Fgfr1, Fgfr4 and KLB were determined. Moreover, to analyze the functionality of the FGF21 signal transduction pathway, the expression levels of Egr-1 and adiponectin were measured. Figure 5 shows that, in scWAT, both the FGF21 and its receptors (FGFR1 and FGFR4) were significantly overexpressed in HFDM mice versus the HFD mice (Figure 5). These data, together with the induction of Egr-1 and adiponectin in scWAT (Figure 5), indicate that maqui supplementation improves FGF21 signaling and effectiveness in scWAT. 

## 4. Discussion

Globally, our data show that maqui dietary supplementation ameliorates the unhealthy effects of HFD. By using diet-induced obese mice, the present study demonstrated that supplemented animals displayed better insulin responsiveness, decreased weight gain and increased thermogenic activity. The analysis of gene expression in different tissues showed that scWAT exhibited differential expression of genes involved in browning, DNL, mitochondrial and peroxisomal FAO, multilocular adipocytes and thermogenesis. These changes were probably related with the increased expression of *Chrebpa, Chrebpb*, *Glut4, Creg1* and *Srebp1c* but also the improvement in FGF21signaling. 

Obesity is essentially caused by an imbalance between energy intake and energy expenditure. Some evidence indicates that, at some point, the white adipose tissue (WAT) fails to adequately keep the surplus of nutrients, which together with an insufficient differentiation of new adipocytes leads to an off-WAT accumulation of ectopic lipids in peripheral relevant organs that may cause the metabolic obesity-related metabolic dysfunctions. It seems obvious that defects in WAT functionality together with peripheral lipotoxicity are key in the onset of metabolic syndrome [61,62,63,64,65,66]. 

The biomedical relevance of brown and beige adipocytes lies in the ability of these cells to increase EE and counteract metabolic diseases such as obesity or type 2 diabetes [1,2]. Indeed, increasing the activity of brown, beige or both fat depots is considered a promising strategy for the treatment of metabolic diseases [67,68].

Berries are an important source of anthocyanins [27,36,69,70]. Anthocyanins are widely distributed water-soluble polyphenols that have shown important health effects including metabolic effects on glucose metabolism [23,24,25,26,27,36,37,69,71,72,73,74,75,76]. In this context, maqui berry features a unique profile of anthocyanidins that includes high amounts of delphinidin-3-O-sambubioside-5-O-glucoside and delphinidin-3-O-sambubioside. These anthocyanins with a sambubioside residue are distinctive of maqui berry since they have been not reported in other edible berries such as blueberries [77], acai [78], blackcurrant, elderberry [79], cranberries [80] or other south Patagonian wild berries [81].

Our results clearly confirm the previously described effects of maqui regarding glucose metabolism but also describe the capacity of maqui to induce browning in HFD-induced obese mice. Dietary-supplemented obese mice showed less weight gain despite their hyperphagic behavior, thus indicating that in some way these animals have a higher metabolic rate, probably due to an increased BAT depot and a more energy consuming scWAT. The absence of effects on other WAT depots indicated that scWAT was the target for maqui beneficial effects against diet-induced obesity and that at least part of the effects of berries on glucose metabolism and insulin sensitivity go through the improvement of adipose tissue functionality.

BAT thermogenesis is stimulated by adrenergic induction of cAMP production, which activates protein kinase A (PKA) to drive transcription of thermogenic genes (including *Ucp1*), lipolysis and FAO. Although less recognized, the activation of BAT thermogenesis paradoxically induces the anabolic DNL pathway [15,82,83,84]. Several studies support the important role of ChREBP isoforms in the control of insulin signaling and lipogenesis in adipose tissue and describe the induction of both isoforms’ activity under the Glut4-dependent glucose uptake [55,85,86,87,88]. In WAT, HFD feeding lowers the expression of *Chrebp* and DNL genes in mice [55]. Moreover, the expression of a constitutively active ChREBP in WAT protects mice against obesity and insulin resistance by among others reducing adiposity and increasing the expression of gene related to adipocyte differentiation and browning [89]. In the same context, an adipose-specific *Chrebp* knockout mice show a decrease in DNL and are insulin resistant with an impaired insulin action in the liver, muscle and fat [85]. Beyond mice, in humans, the expression of *Chrebp* and lipogenic genes in WAT shows a strong correlation with insulin sensitivity, and the improvement of insulin sensitivity in insulin-resistant people restores *Chrebp* and glucose transporter type 4 (*Glut4*) expression in adipose tissue [56].

Our key finding is that this characteristic metabolic feature of BAT also appears in the scWAT of obese mice fed a maqui-supplemented HFD where there is an induction of *Chrebpa*, *Chrebpb* and *Glut4* expression and also higher mRNA levels of genes involved in DNL, multilocular LDs formation and thermogenesis/browning. The remaining question is how ChREBP is activated under maqui supplementation. The increased levels of Glut4 in HFDM mice indicated that glucose or glucose metabolites could be the major inducers of Chrebp expression [90,91]. Moreover, although ChREBP was initially identified as a glucose-responsive factor, recent evidence suggests that it is also essential for fructose-induced lipogenesis both in the small intestine and liver [55,92]. The effects of maqui could, therefore, be attributed to its fructose content. However, the absence of Chrebpb induction in liver (data not shown) allows us to rule out this possibility.

SREBP1c is also a key regulator of hepatic DNL [93,94,95,96,97,98]. It has been demonstrated that, in the liver, SREBP1c and ChREBP are both necessary for the expression of lipogenic and glycolytic genes [88,99] In adipose tissue, the role of SREPB1c is more controversial. In adipose tissue, the mRNA levels of lipogenic genes did not change in animals using a *Srebp1c* loss of function or gain of function approach, thus indicating that in this tissues *Srebp1c* is not essential for DNL activation [58]. By contrast, mice under caloric restriction (CR) showed an SREBP1c/PGC1a-dependant induction of DNL in adipose tissue giving to *Srebp1c* an role on activating this metabolic pathway under this specific nutritional condition [100]. Finally, CREG1 has been described as an inducer of Ucp1 and FGF21 expression in an adipocyte P2–Creg1-transgenic (Tg) mice and globally of BAT adipogenesis and browning [101,102]. Our results indicate that maqui supplementation is able to induce the expression of *Chrebpb, Srebp1c, Pgc1a* and *Creg1*, thus we cannot discard any of them as possible contributors to the induction of DNL and thermogenic genes observed.

In addition, in the scWAT of dietary-supplemented obese mice, there was an increase in the expression of peroxisomal FAO enzymes that would make possible the contradiction of having FAO and DNL simultaneously active. Peroxisomal FAO is important in this scenario where, despite an increased expression of *Cpt1b*, the rate-limiting enzyme of mitochondrial FAO, this pathway would be inhibited by the malonyl-CoA produced by DNL.

Apart from the abovementioned mechanisms our data also pointed out the improvement of FGF21 signaling as a way through which maqui could exert its beneficial effects. It has been widely described that FGF21 levels are increased in obesity and diabetes in both animal models and humans [60,103,104]. The downregulation of FGF21 receptors in adipose tissue seems to be the key point to explain the FGF21-resistant state described mainly in obese mice as well as, in some studies, in humans [43,59,60,105,106,107,108]. In this context, the restoring of FGF21 signaling can be considered as a potential therapeutic strategy to improve the metabolic parameters of obese individuals and to reduce the risk of obesity-related diseases and some evidence support this hypothesis [7,109]. In various rodent models of diet-induced obesity a positive correlation between the beneficial effects of polyphenol-rich fruit extracts and FGF21 has been described. This correlation with FGF21 can be due to an induction of FGF21 levels [110,111] or an improvement of the FGF21 signaling [7,112,113]. Finally, FGF21-resitance in adipose tissue has been linked to a decreased production of adiponectin [114]. Adiponectin induces fatty acid oxidation leading to a reduction of ectopic lipids and finally the improvement of insulin sensitivity [115]. In our results, the induction of the mRNA levels of *adiponectin*, *FgfR1*, *FgfR4* and *Egr1* in scWAT of HFDM mice indicates that, in obesity, maqui supplementation increases the sensitivity to FGF21 of scWAT.

Although our results do not allow us to discard that other signaling pathways could stimulate the white to beige/brown transition described, the improvement of FGF21 signaling together with the overexpression of ChREBP, CREG1, PGC1a and SREBP1c are at least key players in the induction of browning described under maqui supplementation. Neuroendocrine signaling or molecules such as leptin or Bmp8b will be analyzed in further studies to try to complete the signaling cascade activated by maqui [2,67,116].

To summarize, we demonstrated that a nutritional intervention with maqui partially alleviates the unhealthy effects of HFD in mice. Our results provide evidence that, in mice, a dietary supplementation with maqui added to beverage activates the induction of fuel storage and thermogenesis characteristic of a brown-like phenotype in scWAT. Finally, based on previously published data, our results indicate that maqui could exert its effects, at least in part, through the induction of *Chrebpb* expression and the improvement of FGF21 signaling. Finally, it is worth mentioning that in this work the dose of maqui was scaled-down from the polyphenol intake recommended as beneficial in humans by the Predimed Study [45,46]. This is important because several phenolic compounds such as resveratrol, quercetin, cyanidin-3-glucoside (C_3_G), capsaicin, hesperidin have green tea extract have been described as inducers of BAT activity or WAT browning but in most cases the dietary supplementation was performed using high doses and only the active compounds [9,10,11,12,13,117,118,119,120,121].

### Limitations of the Present Work and Further Studies

This work clearly demonstrates that maqui is effective in obese mice. The impact of maqui in healthy individual needs to be evaluated. We do not have data about the effects of maqui on normal chow-fed animals where neither obesity nor insulin resistant is present, but this experimental approach will be included in our further studies. Positive results in this follow-up experiment will allow pointing out the efficacy of the consumption of maqui in the prevention of some metabolic diseases and whether to include its regular consumption as part of a heathy dietary pattern.

On the other hand, despite the observed changes in gene expression, together with the scWAT and BAT appearance and the histological analysis to define properly the browning phenotype, in this study, the translation to protein was assumed. To overcome this limitation, Western blot analyses will be performed to reinforce our results and deepen the mechanism of action of maqui.

## 5. Conclusions

In conclusion, our data provide evidence that, in obese mice, a dietary intervention with a regular dose of an anthocyanidin-enriched berry (maqui) can induce a browning phenotype in scWAT and improve partially the insulin sensitivity, thus ameliorating some of the unhealthy effects of HFD. These effects reinforce the anthocyanidin-enriched foods as a potential strategy to prevent or treat type 2 diabetes and obesity-related diseases and point out maqui as a putative functional fruit to counteract at least in part obesity and its metabolic complications. The data presented in this manuscript reinforce the inclusion of maqui in the diet of obese individuals.

## Figures and Tables

**Table 1:** Anthocyanidin composition of the lyophilized maqui berry. Anthocyanins were determined by UPLC-DAD. The table shows the concentration in mg/g and mmol/kg of the different anthocyanidins detected. The table includes the retention time (min), the limit of detection (LOD) and the limit of quantification (LOQ) for each molecule.

Compound	Conc. (mg/g)	Conc. (mmol/kg)	Retention Time (min)	LOD(mg/L)	LOQ(mg/L)
Delphinidin-3-O-sambubioside-5-O-glucoside	19.645 ± 0.788	24.71 ± 0.99	3.5	1.81	6.02
Delphinidin-3-O-sambubioside	17.770 ± 1.178	28.07 ± 1.86	5.	0.30	1.00
Cyanidin-3-O-sambubioside-5-O-glucoside	2.447 ± 0.063	3.14 ± 0.08	5.5	0.75	2.50
Not identified (quantified as cyd-3-0-glu)	0.402 ± 0.050	0.83 ± 0.10	6.5	-	-
Cyanidin-3-O-glucoside	2.148 ± 0.158	4.43 ± 0.33	7	0.11	0.35
Cyanidin-3-O-sambubioside	2.642 ± 0.201	4.28 ± 0.33	7.3	0.17	0.56
TOTAL	45.052	65.46			

## Supplementary Materials

The following are available online at https://www.mdpi.com/2076-3921/8/9/360/s1, Table S1: Maqui extract nutritional composition, Table S2: Sequences of the primers used in SYBR Green assays and references of the probes used in Taqman assays, Figure S1: Chromatogram of the anthocyanins identified in maqui samples. 1:Delphinidin-3-O-sambubioside-5-O-glucoside;2:Delphinidin-3-O-sambubioside; 3:Cyanidin-3-O-sambubioside-5-O-glucoside; 4: Unknown; 5: Cyanidin-3-O-glucoside; 6: Cyanidin-3-O-sambubioside.

## References

[1] Townsend K.L., Tseng Y.-H. (2015). Of mice and men: Novel insights regarding constitutive and recruitable brown adipocytes. Int. J. Obes. Suppl..

[2] Harms M., Seale P. (2013). Brown and beige fat: Development, function and therapeutic potential. Nat. Med..

[3] Rosenwald M., Wolfrum C. (2014). The origin and definition of brite versus white and classical brown adipocytes. Adipocyte.

[4] Lshibashi J., Seale P. (2010). Beige can be slimming. Science.

[5] Barbatelli G., Murano I., Madsen L., Hao Q., Jimenez M., Kristiansen K., Giacobino J.P., De Matteis R., Cinti S. (2010). The emergence of cold-induced brown adipocytes in mouse white fat depots is determined predominantly by white to brown adipocyte transdifferentiation. AJP Endocrinol. Metab..

[6] Pérez-Martí A., Garcia-Guasch M., Tresserra-Rimbau A., Carrilho-Do-Rosário A., Estruch R., Salas-Salvadó J., Martínez-González M.Á., Lamuela-Raventós R., Marrero P.F., Haro D. (2017). A low-protein diet induces body weight loss and browning of subcutaneous white adipose tissue through enhanced expression of hepatic fibroblast growth factor 21 (FGF21). Mol. Nutr. Food Res..

[7] Sandoval V., Rodríguez-Rodríguez R., Martínez-Garza Ú., Rosell-Cardona C., Lamuela-Raventós R.M., Marrero P.F., Haro D., Relat J. (2018). Mediterranean Tomato-Based Sofrito Sauce Improves Fibroblast Growth Factor 21 (FGF21) Signaling in White Adipose Tissue of Obese ZUCKER Rats. Mol. Nutr. Food Res..

[8] Vitali A., Murano I., Zingaretti M.C., Frontini A., Ricquier D., Cinti S. (2012). The adipose organ of obesity-prone C57BL/6J mice is composed of mixed white and brown adipocytes. J. Lipid Res..

[9] Arias N., Picó C., Teresa Macarulla M., Oliver P., Miranda J., Palou A., Portillo M.P. (2017). A combination of resveratrol and quercetin induces browning in white adipose tissue of rats fed an obesogenic diet. Obesity.

[10] Serrano A., Asnani-Kishnani M., Rodríguez A.M., Palou A., Ribot J., Bonet M.L. (2018). Programming of the Beige Phenotype in White Adipose Tissue of Adult Mice by Mild Resveratrol and Nicotinamide Riboside Supplementations in Early Postnatal Life. Mol. Nutr. Food Res..

[11] Neyrinck A.M., Bindels L.B., Geurts L., Van Hul M., Cani P.D., Delzenne N.M. (2017). A polyphenolic extract from green tea leaves activates fat browning in high-fat-diet-induced obese mice. J. Nutr. Biochem..

[12] Mosqueda-Solís A., Sánchez J., Portillo M.P., Palou A., Picó C. (2018). Combination of capsaicin and hesperidin reduces the effectiveness of each compound to decrease the adipocyte size and to induce browning features in adipose tissue of western diet fed rats. J. Agric. Food Chem..

[13] Reynés B., Palou M., Rodríguez A.M., Palou A. (2019). Regulation of Adaptive Thermogenesis and Browning by Prebiotics and Postbiotics. Front. Physiol..

[14] Garcia-Ruiz E., Reynes B., Diaz-Rua R., Ceresi E., Oliver P., Palou A. (2015). The intake of high-fat diets induces the acquisition of brown adipocyte gene expression features in white adipose tissue. Int. J. Obes..

[15] Sanchez-Gurmaches J., Tang Y., Jespersen N.Z., Wallace M., Martinez Calejman C., Gujja S., Li H., Edwards Y.J.K., Wolfrum C., Metallo C.M. (2018). Brown Fat AKT2 Is a Cold-Induced Kinase that Stimulates ChREBP-Mediated De Novo Lipogenesis to Optimize Fuel Storage and Thermogenesis. Cell Metab..

[16] Fisher F.M., Maratos-Flier E. (2016). Understanding the Physiology of FGF21. Annu. Rev. Physiol..

[17] Gimeno R.E., Moller D.E. (2014). FGF21-based pharmacotherapy - potential utility for metabolic disorders. Trends Endocrinol. Metab..

[18] De Sousa-Coelho A.L., Relat J., Hondares E., Pérez-Martí A., Ribas F., Villarroya F., Marrero P.F., Haro D. (2013). FGF21 mediates the lipid metabolism response to amino acid starvation. J. Lipid Res..

[19] Fisher F.F., Kleiner S., Douris N., Fox E.C., Mepani R.J., Verdeguer F., Wu J., Kharitonenkov A., Flier J.S., Maratos-Flier E. (2012). FGF21 regulates PGC-1α and browning of white adipose tissues in adaptive thermogenesis. Genes Dev..

[20] Lin Z., Tian H., Lam K.S.L., Lin S., Hoo R.C.L., Konishi M., Itoh N., Wang Y., Bornstein S.R., Xu A. (2013). Adiponectin mediates the metabolic effects of FGF21 on glucose homeostasis and insulin sensitivity in mice. Cell Metab..

[21] Lin Z., Pan X., Wu F., Ye D., Zhang Y., Wang Y., Jin L., Lian Q., Huang Y., Ding H. (2015). Fibroblast growth factor 21 prevents atherosclerosis by suppression of hepatic sterol regulatory element-binding protein-2 and induction of adiponectin in mice. Circulation.

[22] Pérez-Martí A., Sandoval V., Marrero P.F., Haro D., Relat J. (2017). Nutritional regulation of fibroblast growth factor 21: From macronutrients to bioactive dietary compounds. Horm. Mol. Biol. Clin. Investig..

[23] Tsuda T. (2012). Dietary anthocyanin-rich plants: Biochemical basis and recent progress in health benefits studies. Mol. Nutr. Food Res..

[24] Tsuda T. (2008). Regulation of adipocyte function by anthocyanins; Possibility of preventing the metabolic syndrome. J. Agric. Food Chem..

[25] He J., Giusti M.M. (2010). Anthocyanins: Natural Colorants with Health-Promoting Properties. Annu. Rev. Food Sci. Technol..

[26] Guo H., Ling W. (2015). The update of anthocyanins on obesity and type 2 diabetes: Experimental evidence and clinical perspectives. Rev. Endocr. Metab. Disord..

[27] Overall J., Bonney S.A., Wilson M., Beermann A., Grace M.H., Esposito D., Lila M.A., Komarnytsky S. (2017). Metabolic effects of berries with structurally diverse anthocyanins. Int. J. Mol. Sci..

[28] Tsuda T. (2016). Recent Progress in Anti-Obesity and Anti-Diabetes Effect of Berries. Antioxidants.

[29] Vendrame S., Del Bo’ C., Ciappellano S., Riso P., Klimis-Zacas D. (2016). Berry Fruit Consumption and Metabolic Syndrome. Antioxidants.

[30] Norberto S., Silva S., Meireles M., Faria A., Pintado M., Calhau C. (2013). Blueberry anthocyanins in health promotion: A metabolic overview. J. Funct. Foods.

[31] Coe S., Ryan L. (2016). Impact of polyphenol-rich sources on acute postprandial glycaemia: A systematic review. J. Nutr. Sci..

[32] Blumberg J.B., Basu A., Krueger C.G., Lila M.A., Neto C.C., Novotny J.A., Reed J.D., Rodriguez-Mateos A., Toner C.D. (2016). Impact of Cranberries on Gut Microbiota and Cardiometabolic Health: Proceedings of the Cranberry Health Research Conference 2015. Adv. Nutr..

[33] Escribano-Bailón M.T., Alcalde-Eon C., Muñoz O., Rivas-Gonzalo J.C., Santos-Buelga C. (2006). Anthocyanins in berries of Maqui (*Aristotelia chilensis* (Mol.) Stuntz). Phytochem. Anal..

[34] Miranda-Rottmann S., Aspillaga A.A., Pérez D.D., Vasquez L., Martinez A.L.F., Leighton F. (2002). Juice and phenolic fractions of the berry Aristotelia chilensis inhibit LDL oxidation in vitro and protect human endothelial cells against oxidative stress. J. Agric. Food Chem..

[35] Watson R.R., Schonlau F. (2015). Nutraceutical and antioxidant effects of a delphinidin-rich maqui berry extract Delphinol (R): A review. Minerva Cardioangiol..

[36] Rojo L.E., Ribnicky D., Logendra S., Poulev A., Rojas-Silva P., Kuhn P., Dorn R., Grace M.H., Lila M.A., Raskin I. (2012). In vitro and in vivo anti-diabetic effects of anthocyanins from Maqui Berry (*Aristotelia chilensis*). Food Chem..

[37] Alvarado J.L., Leschot A., Olivera-Nappa Á., Salgado A.M., Rioseco H., Lyon C., Vigil P. (2016). Delphinidin-rich maqui berry extract (Delphinol^®^) lowers fasting and postprandial glycemia and insulinemia in prediabetic individuals during oral glucose tolerance tests. Biomed. Res. Int..

[38] Wang Y., Rimm E.B., Stampfer M.J., Willett W.C., Hu F.B. (2005). Comparison of abdominal adiposity and overall obesity in predicting risk of type 2 diabetes among men. Am. J. Clin. Nutr..

[39] McLaughlin T., Lamendola C., Liu A., Abbasi F. (2011). Preferential fat deposition in subcutaneous versus visceral depots is associated with insulin sensitivity. J. Clin. Endocrinol. Metab..

[40] Snijder M.B., Dekker J.M., Visser M., Bouter L.M., Stehouwer C.D.A., Kostense P.J., Yudkin J.S., Heine R.J., Nijpels G., Seidell J.C. (2003). Associations of hip and thigh circumferences independent of waist circumference with the incidence of type 2 diabetes: The Hoorn Study. Am. J. Clin. Nutr..

[41] Tran T.T., Yamamoto Y., Gesta S., Kahn C.R. (2008). Beneficial Effects of Subcutaneous Fat Transplantation on Metabolism. Cell Metab..

[42] Stefan N., Schick F., Häring H.U. (2017). Causes, Characteristics, and Consequences of Metabolically Unhealthy Normal Weight in Humans. Cell Metab..

[43] Li H., Wu G., Fang Q., Zhang M., Hui X., Sheng B., Wu L., Bao Y., Li P., Xu A. (2018). Fibroblast growth factor 21 increases insulin sensitivity through specific expansion of subcutaneous fat. Nat. Commun..

[44] Andres-Lacueva C., Shukitt-Hale B., Galli R.L., Jauregui O., Lamuela-Raventos R.M., Joseph J.A. (2005). Anthocyanins in aged blueberry-fed rats are found centrally and may enhance memory. Nutr. Neurosci..

[45] Tresserra-Rimbau A., Medina-Remón A., Pérez-Jiménez J., Martínez-González M.A., Covas M.I., Corella D., Salas-Salvadó J., Gómez-Gracia E., Lapetra J., Arós F. (2013). Dietary intake and major food sources of polyphenols in a Spanish population at high cardiovascular risk: The PREDIMED study. Nutr. Metab. Cardiovasc. Dis..

[46] Tresserra-Rimbau A., Guasch-Ferre M., Salas-Salvado J., Toledo E., Corella D., Castaner O., Guo X., Gomez-Gracia E., Lapetra J., Aros F. (2016). Intake of Total Polyphenols and Some Classes of Polyphenols Is Inversely Associated with Diabetes in Elderly People at High Cardiovascular Disease Risk. J. Nutr..

[47] Fredes C., Osorio M.J., Parada J., Robert P. (2018). Stability and bioaccessibility of anthocyanins from maqui (*Aristotelia chilensis* [Mol.] Stuntz) juice microparticles. LWT Food Sci. Technol..

[48] Brauch J.E., Reuter L., Conrad J., Vogel H., Schweiggert R.M., Carle R. (2017). Characterization of anthocyanins in novel Chilean maqui berry clones by HPLC–DAD–ESI/MSn and NMR-spectroscopy. J. Food Compos. Anal..

[49] Genskowsky E., Puente L.A., Pérez-Álvarez J.A., Fernández-López J., Muñoz L.A., Viuda-Martos M. (2016). Determination of polyphenolic profile, antioxidant activity and antibacterial properties of maqui [*Aristotelia chilensis* (Molina) Stuntz] a Chilean blackberry. J. Sci. Food Agric..

[50] Lucas-Gonzalez R., Navarro-Coves S., Pérez-Álvarez J.A., Fernández-López J., Muñoz L.A., Viuda-Martos M. (2016). Assessment of polyphenolic profile stability and changes in the antioxidant potential of maqui berry (*Aristotelia chilensis* (Molina) Stuntz) during in vitro gastrointestinal digestion. Ind. Crops Prod..

[51] Prior R.L.E., Wilkes S.R., Rogers T., Khanal R.C., Wu X., Howard L.R. (2010). Purified blueberry anthocyanins and blueberry juice alter development of obesity in mice fed an obesogenic high-fat diet. J. Agric. Food Chem..

[52] Syamaladevi R.M., Sablani S.S., Tang J., Powers J., Swanson B.G. (2011). Stability of Anthocyanins in Frozen and Freeze-Dried Raspberries during Long-Term Storage: In Relation to Glass Transition. J. Food Sci..

[53] Nishimoto Y., Tamori Y. (2017). CIDE Family-Mediated Unique Lipid Droplet Morphology in White Adipose Tissue and Brown Adipose Tissue Determines the Adipocyte Energy Metabolism. J. Atheroscler. Thromb..

[54] Nishimoto Y., Nakajima S., Tateya S., Saito M., Ogawa W., Tamori Y. (2017). Cell death-inducing DNA fragmentation factor A-like effector A and fat-specific protein 27β coordinately control lipid droplet size in brown adipocytes. J. Biol. Chem..

[55] Herman M.A., Peroni O.D., Villoria J., Schön M.R., Abumrad N.A., Blüher M., Klein S., Kahn B.B. (2012). A novel ChREBP isoform in adipose tissue regulates systemic glucose metabolism. Nature.

[56] Eissing L., Scherer T., Tödter K., Knippschild U., Greve J.W., Buurman W.A., Pinnschmidt H.O., Rensen S.S., Wolf A.M., Bartelt A. (2013). De novo lipogenesis in human fat and liver is linked to ChREBP-β and metabolic health. Nat. Commun..

[57] Kursawe R., Caprio S., Giannini C., Narayan D., Lin A., D’Adamo E., Shaw M., Pierpont B., Cushman S.W., Shulman G.I. (2013). Decreased transcription of ChREBP-α/β isoforms in abdominal subcutaneous adipose tissue of obese adolescents with prediabetes or early type 2 diabetes: Associations with insulin resistance and hyperglycemia. Diabetes.

[58] Song Z., Xiaoli A., Yang F., Song Z., Xiaoli A.M., Yang F. (2018). Regulation and Metabolic Significance of De Novo Lipogenesis in Adipose Tissues. Nutrients.

[59] Fisher F.M., Chui P.C., Antonellis P.J., Bina H.A., Kharitonenkov A., Flier J.S., Maratos-Flier E. (2010). Obesity is a fibroblast growth factor 21 (FGF21)-resistant state. Diabetes.

[60] Zhang X., Yeung D.C.Y., Karpisek M., Stejskal D., Zhou Z., Liu F., Wong R.L.C., Chow W., Tso A.W.K., Lam K.S.L. (2008). Serum FGF21 Levels Are Increased in Obesity and Are in Humans. Diabetes.

[61] Montague C.T., O’Rahilly S. (2000). The perils of portliness: Causes and consequences of visceral adiposity. Diabetes.

[62] Peirce V., Carobbio S., Vidal-Puig A. (2014). The different shades of fat. Nature.

[63] Carobbio S., Rodriguez-Cuenca S., Vidal-Puig A. (2011). Origins of metabolic complications in obesity: Ectopic fat accumulation. the importance of the qualitative aspect of lipotoxicity. Curr. Opin. Clin. Nutr. Metab. Care.

[64] Virtue S., Vidal-Puig A. (2010). Adipose tissue expandability, lipotoxicity and the Metabolic Syndrome—An allostatic perspective. Biochim. Biophys. Acta Mol. Cell Biol. Lipids.

[65] Boden G., Shulman G.I. (2002). Free fatty acids in obesity and type 2 diabetes: Defining their role in the development of insulin resistance and β-cell dysfunction. Eur. J. Clin. Investig..

[66] Unger R.H. (2002). Lipotoxic Diseases. Annu. Rev. Med..

[67] Whittle A., Relat-Pardo J., Vidal-Puig A. (2013). Pharmacological strategies for targeting BAT thermogenesis. Trends Pharmacol. Sci..

[68] Bartelt A., Heeren J. (2014). Adipose tissue browning and metabolic health. Nat. Rev. Endocrinol..

[69] Skates E., Overall J., DeZego K., Wilson M., Esposito D., Lila M.A., Komarnytsky S. (2018). Berries containing anthocyanins with enhanced methylation profiles are more effective at ameliorating high fat diet-induced metabolic damage. Food Chem. Toxicol..

[70] Skrovankova S., Sumczynski D., Mlcek J., Jurikova T., Sochor J. (2015). Bioactive compounds and antioxidant activity in different types of berries. Int. J. Mol. Sci..

[71] de Pascual-Teresa S., Moreno D.A., García-Viguera C. (2010). Flavanols and anthocyanins in cardiovascular health: A review of current evidence. Int. J. Mol. Sci..

[72] Lee Y.M., Yoon Y., Yoon H., Park H.M., Song S., Yeum K.J. (2017). Dietary anthocyanins against obesity and inflammation. Nutrients.

[73] Valenti L., Riso P., Mazzocchi A., Porrini M., Fargion S., Agostoni C. (2013). Dietary anthocyanins as nutritional therapy for nonalcoholic fatty liver disease. Oxid. Med. Cell. Longev..

[74] Castro-Barquero S., Lamuela-Raventós R.M., Doménech M., Estruch R. (2018). Relationship between mediterranean dietary polyphenol intake and obesity. Nutrients.

[75] Wu T., Yu Z., Tang Q., Song H., Gao Z., Chen W., Zheng X. (2013). Honeysuckle anthocyanin supplementation prevents diet-induced obesity in C57BL/6 mice. Food Funct..

[76] Wallace T.C. (2011). Anthocyanins in Cardiovascular Disease. Adv. Nutr..

[77] Grace M.H., Ribnicky D.M., Kuhn P., Poulev A., Logendra S., Yousef G.G., Raskin I., Lila M.A. (2009). Hypoglycemic activity of a novel anthocyanin-rich formulation from lowbush blueberry, Vaccinium angustifolium Aiton. Phytomedicine.

[78] Fibigr J., Šatínský D., Solich P. (2017). A UHPLC method for the rapid separation and quantification of anthocyanins in acai berry and dry blueberry extracts. J. Pharm. Biomed. Anal..

[79] Schreckinger M.E., Wang J., Yousef G., Lila M.A., De Mejia E.G. (2010). Antioxidant capacity and in Vitro inhibition of adipogenesis and inflammation by phenolic extracts of Vaccinium floribundum and Aristotelia chilensis. J. Agric. Food Chem..

[80] Milbury P.E., Vita J.A., Blumberg J.B. (2010). Anthocyanins are Bioavailable in Humans following an Acute Dose of Cranberry Juice. J. Nutr..

[81] Ruiz A., Hermosín-Gutiérrez I., Vergara C., von Baer D., Zapata M., Hitschfeld A., Obando L., Mardones C. (2013). Anthocyanin profiles in south Patagonian wild berries by HPLC-DAD-ESI-MS/MS. Food Res. Int..

[82] Mottillo E.P., Balasubramanian P., Lee Y.-H., Weng C., Kershaw E.E., Granneman J.G. (2014). Coupling of lipolysis and de novo lipogenesis in brown, beige, and white adipose tissues during chronic β3-adrenergic receptor activation. J. Lipid Res..

[83] Yu X.X., Lewin D.A., Forrest W., Adams S.H. (2002). Cold elicits the simultaneous induction of fatty acid synthesis and β-oxidation in murine brown adipose tissue: Prediction from differential gene expression and confirmation in vivo. FASEB J..

[84] Bartelt A., Weigelt C., Cherradi M.L., Niemeier A., Tödter K., Heeren J., Scheja L. (2013). Effects of adipocyte lipoprotein lipase on de novo lipogenesis and white adipose tissue browning. Biochim. Biophys. Acta Mol. Cell Biol. Lipids.

[85] Vijayakumar A., Aryal P., Wen J., Syed I., Vazirani R.P., Moraes-Vieira P.M., Camporez J.P., Gallop M.R., Perry R.J., Peroni O.D. (2017). Absence of Carbohydrate Response Element Binding Protein in Adipocytes Causes Systemic Insulin Resistance and Impairs Glucose Transport. Cell Rep..

[86] Katz L.S., Xu S., Ge K., Scott D.K., Gershengorn M.C. (2018). T_3_ and glucose coordinately stimulate ChREBP-Mediated Ucp1 expression in brown adipocytes from male mice. Endocrinology.

[87] Witte N., Muenzner M., Rietscher J., Knauer M., Heidenreich S., Nuotio-Antar A.M., Graef F.A., Fedders R., Tolkachov A., Goehring I. (2015). The glucose sensor ChREBP links *de-novo* lipogenesis to PPARγ activity and adipocyte differentiation. Endocrinology.

[88] Linden A.G., Li S., Choi H.Y., Fang F., Fukasawa M., Uyeda K., Hammer R.E., Horton J.D., Engelking L.J., Liang G. (2018). Interplay between ChREBP and SREBP-1c coordinates postprandial glycolysis and lipogenesis in livers of mice. J. Lipid Res..

[89] Nuotio-Antar A.M., Poungvarin N., Li M., Schupp M., Mohammad M., Gerard S., Zou F., Chan L. (2015). FABP4-cre mediated expression of constitutively active ChREBP protects against obesity, fatty liver, and insulin resistance. Endocrinology.

[90] Filhoulaud G., Guilmeau S., Dentin R., Girard J., Postic C. (2013). Novel insights into ChREBP regulation and function. Trends Endocrinol. Metab..

[91] Baraille F., Planchais J., Dentin R., Guilmeau S., Postic C. (2015). Integration of ChREBP-Mediated Glucose Sensing into Whole Body Metabolism. Physiology.

[92] Lee H.J., Cha J.Y. (2018). Recent insights into the role of ChREBP in intestinal fructose absorption and metabolism. BMB Rep..

[93] Kohjima M., Higuchi N., Kato M., Kotoh K., Yoshimoto T., Fujino T., Yada M., Yada R., Harada N., Enjoji M. (2008). SREBP-1c, regulated by the insulin and AMPK signaling pathways, plays a role in nonalcoholic fatty liver disease. Int. J. Mol. Med..

[94] Shimomura I., Bashmakov Y., Horton J.D. (1999). Increased levels of nuclear SREBP-1c associated with fatty livers in two mouse models of diabetes mellitus. J. Biol. Chem..

[95] Knebel B., Haas J., Hartwig S., Jacob S., Köllmer C., Nitzgen U., Muller-Wieland D., Kotzka J. (2012). Liver-specific expression of transcriptionally active srebp-1c is associated with fatty liver and increased visceral fat mass. PLoS ONE.

[96] Yahagi N., Shimano H., Hasty A.H., Matsuzaka T., Ide T., Yoshikawa T., Amemiya-Kudo M., Tomita S., Okazaki H., Tamura Y. (2002). Absence of sterol regulatory element-binding protein-1 (SREBP-1) ameliorates fatty livers but not obesity or insulin resistance in Lepob/Lepob mice. J. Biol. Chem..

[97] Moon Y.A., Liang G., Xie X., Frank-Kamenetsky M., Fitzgerald K., Koteliansky V., Brown M.S., Goldstein J.L., Horton J.D. (2012). The Scap/SREBP pathway is essential for developing diabetic fatty liver and carbohydrate-induced hypertriglyceridemia in animals. Cell Metab. Cell Metab..

[98] Kim J.B., Spiegelman B.M. (1996). ADD1/SREBP1 promotes adipocyte differentiation and gene expression linked to fatty acid metabolism. Genes Dev..

[99] Gnoni A., Siculella L., Paglialonga G., Damiano F., Giudetti A.M. (2019). 3,5-diiodo-L-thyronine increases de novo lipogenesis in liver from hypothyroid rats by SREBP-1 and ChREBP-mediated transcriptional mechanisms. IUBMB Life.

[100] Kobayashi M., Fujii N., Narita T., Higami Y., Kobayashi M., Fujii N., Narita T., Higami Y. (2018). SREBP-1c-Dependent Metabolic Remodeling of White Adipose Tissue by Caloric Restriction. Int. J. Mol. Sci..

[101] Kusudo T., Hashimoto M., Kataoka N., Li Y., Nozaki A., Yamashita H. (2019). CREG1 promotes uncoupling protein 1 expression and brown adipogenesis in vitro. J. Biochem..

[102] Hashimoto M., Kusudo T., Takeuchi T., Kataoka N., Mukai T., Yamashita H. (2019). CREG1 stimulates brown adipocyte formation and ameliorates diet-induced obesity in mice. FASEB J..

[103] Mraz M., Bartlova M., Lacinova Z., Michalsky D., Kasalicky M., Haluzikova D., Matoulek M., Dostalova I., Humenanska V., Haluzik M. (2009). Serum concentrations and tissue expression of a novel endocrine regulator fibroblast growth factor-21 in patients with type 2 diabetes and obesity. Clin. Endocrinol..

[104] Dushay J., Chui P.C., Gopalakrishnan G.S., Varela-Rey M., Crawley M., Fisher F.M., Badman M.K., Martinez-Chantar M.L., Maratos-Flier E. (2010). Increased fibroblast growth factor 21 in obesity and nonalcoholic fatty liver disease. Gastroenterology.

[105] Ge X., Chen C., Hui X., Wang Y., Lam K.S.L., Xu A. (2011). Fibroblast growth factor 21 induces glucose transporter-1 expression through activation of the serum response factor/Ets-like protein-1 in adipocytes. J. Biol. Chem..

[106] Samms R.J., Cheng C.C., Kharitonenkov A., Gimeno R.E., Adams A.C. (2016). Overexpression of β-klotho in adipose tissue sensitizes male mice to endogenous FGF21 and provides protection from diet-induced obesity. Endocrinology.

[107] Gallego-Escuredo J.M., Gómez-Ambrosi J., Catalan V., Domingo P., Giralt M., Frühbeck G., Villarroya F. (2015). Opposite alterations in FGF21 and FGF19 levels and disturbed expression of the receptor machinery for endocrine FGFs in obese patients. Int. J. Obes..

[108] Nygaard E.B., Møller C.L., Kievit P., Grove K.L., Andersen B. (2014). Increased fibroblast growth factor 21 expression in high-fat diet-sensitive non-human primates (*Macaca mulatta*). Int. J. Obes..

[109] Geng L., Liao B., Jin L., Huang Z., Triggle C.R., Ding H., Zhang J., Huang Y., Lin Z., Xu A. (2019). Exercise Alleviates Obesity-Induced Metabolic Dysfunction via Enhancing FGF21 Sensitivity in Adipose Tissues. Cell Rep..

[110] Monika P., Geetha A. (2015). The modulating effect of Persea americana fruit extract on the level of expression of fatty acid synthase complex, lipoprotein lipase, fibroblast growth factor-21 and leptin–A biochemical study in rats subjected to experimental hyperlipidemia and obesit. Phytomedicine.

[111] Tian L., Zeng K., Shao W., Yang B.B., Fantus I.G., Weng J., Jin T. (2015). Short-Term Curcumin Gavage Sensitizes Insulin Signaling in Dexamethasone-Treated C57BL/6 Mice. J. Nutr..

[112] Song H., Zheng Z., Wu J., Lai J., Chu Q., Zheng X. (2016). White Pitaya (*Hylocereus undatus*) Juice Attenuates Insulin Resistance and Hepatic Steatosis in Diet-Induced Obese Mice. PLoS ONE.

[113] Yu Y., Zhang X.H., Ebersole B., Ribnicky D., Wang Z.Q. (2013). Bitter melon extract attenuating hepatic steatosis may be mediated by FGF21 and AMPK/Sirt1 signaling in mice. Sci. Rep..

[114] Hui X., Feng T., Liu Q., Gao Y., Xu A. (2016). The FGF21-adiponectin axis in controlling energy and vascular homeostasis. J. Mol. Cell Biol..

[115] Yoon M.J., Lee G.Y., Chung J.J., Ahn Y.A., Hong S.H., Kim J.B. (2006). Adiponectin increases fatty acid oxidation in skeletal muscle cells by sequential activation of AMP-activated protein kinase, p38 mitogen-activated protein kinase, and peroxisome proliferator-activated receptor α. Diabetes.

[116] de Cruz Rodrigues K.C., Pereira R.M., de Campos T.D.P., de Moura R.F., de Silva A.S.R., Cintra D.E., Ropelle E.R., Pauli J.R., de Araújo M.B., de Moura L.P. (2018). The Role of Physical Exercise to Improve the Browning of White Adipose Tissue via POMC Neurons. Front. Cell. Neurosci..

[117] Alberdi G., Rodríguez V.M., Macarulla M.T., Miranda J., Churruca I., Portillo M.P. (2013). Hepatic lipid metabolic pathways modified by resveratrol in rats fed an obesogenic diet. Nutrition.

[118] You Y., Liang C., Han X., Guo J., Ren C., Liu G., Huang W., Zhan J. (2017). Mulberry anthocyanins, cyanidin 3-glucoside and cyanidin 3-rutinoside, increase the quantity of mitochondria during brown adipogenesis. J. Funct. Foods.

[119] You Y., Yuan X., Liu X., Liang C., Meng M., Huang Y., Han X., Guo J., Guo Y., Ren C. (2017). Cyanidin-3-glucoside increases whole body energy metabolism by upregulating brown adipose tissue mitochondrial function. Mol. Nutr. Food Res..

[120] You Y., Yuan X., Lee H.J., Huang W., Jin W., Zhan J. (2015). Mulberry and mulberry wine extract increase the number of mitochondria during brown adipogenesis. Food Funct..

[121] Anhê F.F., Nachbar R.T., Varin T.V., Trottier J., Dudonné S., Le Barz M., Feutry P., Pilon G., Barbier O., Desjardins Y. (2019). Treatment with camu camu (*Myrciaria dubia*) prevents obesity by altering the gut microbiota and increasing energy expenditure in diet-induced obese mice. Gut.

