# Supplementary material for: Lyophilized Maqui (Aristotelia chilensis) Berry Induces Browning in the Subcutaneous White Adipose Tissue and Ameliorates the Insulin Resistance in High Fat Diet-Induced Obese Mice
Source: Antioxidants (Basel). 2019 Sep 1;8(9):360. doi: 10.3390/antiox8090360 (PMC6769892; doi:10.3390/antiox8090360)
Supplement: Supplementary file 1 [file antioxidants-08-00360-s001.pdf]

Table S1: Nutritional composition of the lyophilized maqui

	**100g**	**Per serving (2g)**
**Calories**	914kJ/232Kcal	19.7kJ/4.64Kcal
**Total Fat**	10.8g	0.2g
**Total Carbohydrate**	78g	1.6g
Sugars	24.2g	0.5g
Dietary fiber	50.4g	1.0g
**Total Protein**	6.2g	0.1g
**Sodium**	20.1mg	0.4mg
**Polyphenols**	6550 mg (GAE)	131 (GAE)

Table S2: Sequences of the primers used in SYBR Green assays and references of the probes used in Taqman assays.

Gene	Forward	Reverse
18s	4310893E – 1510059 (Taqman)	
mFgf21	Mm0840165_g1 (Taqman)	
mB2M	F-5`-ACTGATACATACGCCTGCAGAGTT -3`	R-5`-TCACATGTCTCGATCCCAGTAGA -3`
mAcox3	F-5`-GCCTCCTTCAACTCTGGGG -3`	R-5`-TCAGTTCTTCGTAGCTTCTCTAGG-3`
mEhhadh	F-5`-AATACAGCGATACCAGAAGCC -3`	R-5`-ATTCCCAGCATCACTTCCG -3`
mGlut4	F-5`-GTGACTGGAACACTGGTCCTA-3`	R-5`-CCAGCCAGTTGCATTGTAG -3`
mAcly	F-5`-GCCAGCGGGAGCACATC -3`	R-5`-CTTTGAGGTGCCACTTCATC -3
mGlyK	F-5`-TGGCAGCCGCGAAGAA-3`	R-5`-TGATGACTAAGAAGTTCAGCTGTT-3`
mAcaca	F-5`-TGTACAAGCAGTGTGGGCTGGCT-3`	R-5`-CCACATGGCCTGGCTTGGAGGG -3`
mFasn	F-5`-GCTGCGGAAACTTCAGGAAAT -3`	R-5`-AGAGACGTGTCACTCCTGGACTT -3`
mChrebpb	F-5`- TCTGCAGATCGGTGGAG-3`	R-5`-CTTGTCCCGGCATAGCAAC -3`
mChrebpa	F-5`-CGACACTCACCCACCTCTTC -3`	R-5`-TTGTTCAGCCGGATCTTGTC -3`
mUcp1	F-5`-CCCGCTGGACACTGCC-3`	R-5`-ACCTAATGGTACTGGAAGCCTGG -3`
mPrdm16	F- 5’-CAGCACGGTGAAGCCATT-3’	R- 5’-GCGTGCATCCGCTTGTG-3’
mPgc1a	F- 5’-AACCACACCCACAGGATCAGA-3’	R- 5’-CTCTTCGCTTTATTGCTCCATGA-3’
mPparg	F- 5’-GCATCAGGCTTCCACTATGGA-3’	R- 5’-AATCGGATGGTTCTTCGGAAA-3’
mDio2	F- 5’-TGCGCTGTGTCTGGAACAG-3’	R- 5’-CTGGAATTGGGAGCATCTTCA-3’
mFsp27b	F-5`-GTGACCACAGCTTGGGTCGGA -3`	R-5`-GGGTCTCCCGGCTGGGCTTA -3`
mFsp27a	F-5`-GCCACGCGGTATTGCCAGGA -3`	R-5`-GGGTCTCCCGGCTGGGCTTA -3`
mDgat1	F-5`-CGACGGCTACTGGGATCTGA-3`	R-5`-CTCAGGATCAGCATCACCA-3`
mSrebp1c	F-5`-GGAGCCATGGATTGCACATT-3`	R-5`-GGCCCGGGAAGTCACTGT-3`
mCreg1	F-5`-CCGTATCTGGGTCTTGGACT-3’	R-5`-CATGAGCCTCCGAAGACACT-3`
mKlb	F-5`-ACACTGTGGGACACAACCTG-3’	R-5`-ATCCAATGGACCCCAAGGT-3`
mFgfR1	F-5`- CTGGCAGCGATACCACCTAC -3’	R-5`-CTGGGGATGTCCAGTAGGGA-3`
mFgfR4	F-5`- CTGCTTTGGGCAAGTGGTTC -3’	R-5`-TGCCAAATCCTTGTCGGAGG-3`
mEgr1	F-5`- GCCGAGCGAACAACCCTAT- -3’	R-5`-ATAACTCGTCTCCACCCATCGC-3`
mAdiponectin	F-5`- CAGTGGATCTGACGACACCAA - -3’	R-5`- TGGGCAGGATTAAGAGGAACA-3`
